# Novel therapeutic agents in clinical development for systemic lupus erythematosus

**DOI:** 10.1186/1741-7015-11-120

**Published:** 2013-05-03

**Authors:** Natasha Jordan, Pamela MK Lutalo, David P D’Cruz

**Affiliations:** 1 Louise Coote Lupus Unit, St Thomas’ Hospital, Westminster Bridge Road, London, SE1 7EH, UK

**Keywords:** Lupus nephritis, B-cell depletion, BLys, T-cell co-stimulation, Interferon-α, SLE

## Abstract

Conventional immunosuppressive therapies have radically transformed patient survival in systemic lupus erythematosus (SLE), but their use is associated with considerable toxicity and a substantial proportion of patients remain refractory to treatment. A more comprehensive understanding of the complexity of SLE immunopathogenesis has evolved over the past decade and has led to the testing of several biologic agents in clinical trials. There is a clear need for new therapeutic agents that overcome these issues, and biologic agents offer exciting prospects as future SLE therapies.

An array of promising new therapies are currently emerging or are under development including B-cell depletion therapies, agents targeting B-cell survival factors, blockade of T-cell co-stimulation and anti-cytokine therapies, such as monoclonal antibodies against interleukin-6 and interferon-α.

## Introduction

Systemic lupus erythematosus (SLE) is a complex autoimmune rheumatic disease, characterized by unpredictable exacerbations and remissions. Clinical manifestations are variable ranging from arthralgia, photosensitivity and the classic ‘butterfly’ rash to internal organ involvement, most notably renal and central nervous system disease [1]. The prevalence of SLE varies significantly in different ethnic groups. SLE is more commonly seen in those of Afro-Caribbean and Asian origin than in Caucasian populations [2]. The overall prevalence of SLE in the UK is approximately 28 per 100,000 head of population, rising to approximately 200 per 100,000 in Afro-Caribbean females [3].

Lupus nephritis remains a major cause of morbidity and mortality in SLE. There have been major improvements in the risk of premature mortality in patients with lupus nephritis [4]. However, despite advances in the clinical management of lupus nephritis in recent decades with earlier diagnosis of disease and optimization of the currently available immunosuppressive regimens, an estimated 10% to 15% of patients progress to end-stage renal disease (ESRD) [5]. The rate of progression to ESRD and the risk of premature mortality is likely to be even higher in patients of Afro-Caribbean descent [6]. A significant proportion of lupus nephritis patients are refractory to conventional immunosuppressive agents and the potential side effects of these therapies remain significant.

A retrospective review of lupus nephritis patients over a 30-year period (1975 to 2005) from a single center showed that five-year mortality decreased by 60% between the first and second decades of the study but remained unchanged over the third decade with rates of 17.2, 7.7 and 4.7%, respectively, after the diagnosis of renal disease [7]. The rate of progression to ESRD also reached a plateau in the third decade. These results suggest that the benefits of conventional immunosuppressive therapies have been maximized and if further advances in SLE outcomes are to be achieved, novel therapeutic targets must be developed [7].

Over the last two decades, there have been tremendous advances in the understanding of the immunopathology of this autoimmune disorder. A variety of novel therapeutic targets have been identified and there have been many clinical trials in patients with SLE in an attempt to translate these new treatments into clinical practice. The results of these studies have been very mixed and there has been a steep learning curve for everyone involved in designing and executing these trials. SLE is a particularly challenging disease to study due to the broad spectrum of clinical manifestations and varying patterns of disease activity. Furthermore, disease specific outcome measures that were developed for use in observational clinical studies were exposed as inadequate when used in therapeutic clinical trials. This has led to the development of a composite outcome measure, the Systemic Lupus Erythematosus Responder Index (SRI), which has become the industry standard for lupus trials [8]. Another theme that has emerged is the excessive use of corticosteroids. Not only are these a major confounder in assessing disease response, it is now recognized that high dose corticosteroids have significant deleterious effects that may contribute to the development of damage and, hence, long term morbidity and premature mortality [9]. Here we describe novel therapeutic strategies under development for the treatment of SLE, which are summarized in Table 1.

**Table 1:** Summary of potential novel therapeutic options and biologics for SLE

**Drug name**	**Target**	**Study participants**	**Mechanism of action**	**Trials**
**Rituximab**	Chimeric anti-CD20 monoclonal antibody	257 SLE patients with moderately-to-severely active disease ( ≥1 BILAG A score or ≥2 BILAG B scores)	B cell apoptosis or lysis	Explorer
		144 patients with class III or class IV lupus nephritis		Lunar
**Belimumab**	Humanized anti-BLyS monoclonal antibody	865 seropositive SLE patients with SELENA-SLEDAI score ≥6. Severe active lupus nephritis and severe CNS disease excluded.	BLyS inhibition blocks soluble BLys and prevents binding to B cell receptor	Bliss-52
		819 seropositive SLE patients with SELENA-SLEDAI score ≥6. Severe active lupus nephritis & severe CNS disease excluded.		Bliss-76
**Blisibimod**	Anti-BLyS antagonist fusion protein	Active SLE	BLyS inhibition blocks soluble BLys and prevents binding to B cell receptor	Pearl-sc
**Atacicept**	Recombinant fusion protein to TACI-Ig	SLE excluding lupus nephritis.	Inhibition of B cell activation by BLys and APRIL	Phase II/III
		Lupus nephritis study discontinued due to reports of increased infection.		
**Epratuzumab**	Humanized anti-CD22 monoclonal antibody	227 moderate to severe SLE patients.	B cell apoptosis	Emblem
		Moderate to severe SLE patients excluding severe renal and neuropsychiatric disease.		Embody
**Abatacept**	CTL4-Ig fusion protein	SLE with polyarthritis, discoid lesions, or pleuritis and/or pericarditis.	Blockade of co-stimulatory interaction of T and B lymphocytes	Phase III
		Data reanalyzed for lupus nephritis		
**Tocilizumab**	Humanized anti-IL6 receptor monoclonal antibody	SLE patients with mild-to-moderate disease activity.	Inhibition of membrane bound and soluble IL-6 receptor	Phase I
**Sifalimumab**	Humanized anti-IFNα monoclonal antibody	Moderately active SLE.	Inhibition of type I IFN signature	Phase II
**Rontalizumab**	A recombinant humanized monoclonal antibody to IFN-α	Moderate to severe non-renal SLE		

### B-cell depletion therapy

Given that autoantibody production is the hallmark of SLE, it is not surprising that B cell depletion therapy is a promising therapeutic option in the management of SLE. The main drug in current clinical practice is rituximab, with other drugs in development including epratuzumab. B cells, including the populations that interact with T cells, play an integral part in the autoimmune pathogenesis of SLE, and it is thought that after B cell depletion, disease activity may be modified and durable disease remission achieved, minimizing the use of other immunosuppressive agents and corticosteroids.

### Rituximab (anti-CD20)

Rituximab is a chimeric anti-CD20 monoclonal antibody that has been used off-license in the management of severe refractory SLE since 2002. The mechanism of action of rituximab involves antibody-dependent cell toxicity (ADCC), complement-dependent cell toxicity (CDC) and direct apoptosis of CD20^+^ B lymphocytes which results in complete B cell depletion [10]. Plasma cells are unaffected by rituximab as they lack the CD20 surface marker.

A recent review of the efficacy of rituximab in the management of SLE patients with biopsy-proven severe lupus nephritis from pooled data in European cohorts (n = 164) reported the clinical efficacy of rituximab in clinical practice [11]. This open-label data, showing that approximately two-thirds of patients previously unresponsive to conventional therapies had clinical benefit, is in contrast to the two randomized controlled clinical trials (RCTs) of rituximab, which did not meet the primary and secondary endpoints set out during the trial design.

The Study to Evaluate the Efficacy and Safety of Rituximab in Patients With Severe Systemic Lupus Erythematosus (EXPLORER) included patients with moderate to severe SLE but excluded lupus nephritis patients (n = 257) [12]. The EXPLORER RCT compared rituximab plus standard immunosuppressive drugs including mycophenolate mofetil (MMF) (n = 169) to placebo plus standard immunosuppressive therapy, with all patients receiving 10 weeks of high dose corticosteroids. Published data report the failure of the EXPLORER trial to show superiority of rituximab or statistically significant differences in clinical activity when the two treatment arms were compared [12]. Closer examination of the data shows that rituximab achieved effective B cell depletion and, in those patients with positive anti-dsDNA antibodies and low complement levels, significant improvements were seen in these parameters in the rituximab treated patients compared to the placebo group.

The Study to Evaluate the Efficacy and Safety of Rituximab in Subjects with ISN/RPS Class III or IV Lupus Nephritis (LUNAR) trial compared rituximab plus MMF to MMF alone for the management of severe proliferative lupus nephritis class III and class IV. The published results did not show superiority of the rituximab combination therapy [13]. As with the EXPLORER study, rituximab therapy achieved B cell depletion as well as improvements in the levels of anti-dsDNA antibodies and complement levels compared to the placebo treated patients. Thus, in both these studies, a biological effect was seen in the rituximab arms that did not translate into a clinical benefit over and above standard therapies.

There are many possible explanations for the failure of the EXPLORER and LUNAR trials such as the relatively short trial duration and high doses of concomitant corticosteroids. Rituximab continues to be used off-label in a select group of patients with severe refractory SLE. This off-license use of rituximab takes into account the potential benefits reported from clinical practice and the possible complications of biologic therapy, such as severe or recurrent infections, adverse drug reactions and the few case reports of progressive multi-focal leuco-encephalopathy (PML) [14,15].

An additional benefit of rituximab induction therapy followed by MMF maintenance therapy for the management of severe proliferative lupus nephritis class III and class IV, is the ability to reduce and eventually withdraw corticosteroid therapy in patients who respond to treatment [16].

A new treatment strategy termed the Rituxilup regimen has been pioneered in a center in the United Kingdom. The Rituxilup regimen avoids the use of concomitant oral corticosteroid therapy after rituximab induction therapy, thereby minimizing the duration of corticosteroid exposure and steroid side-effects [17]. A proposed randomized controlled trial will be of great clinical relevance in ascertaining the clinical effectiveness, benefits and consequences of this steroid-sparing regimen.

RING – Rituximab for Lupus Nephritis With Remission as a Goal, an investigator-initiated randomized international open multicenter study, aims to determine the clinical effectiveness of rituximab in achieving complete renal remission in lupus nephritis patients with persistent proteinuria (≥1 grams/day) despite a minimum of six months of standard immunosuppressive therapy (http://www.clinicaltrials.gov). This study is still at the development stage.

### Epratuzumab (anti-CD22)

Epratuzumab is an anti-CD22 monoclonal antibody that is currently under investigation for the management of moderate to severe SLE and shows great promise.

CD22 is a B cell specific trans-membrane sialo-glycoprotein which is present on the cell surface of mature naive B cells and transitional B cells but not present on memory B cells or plasma cells [18]. CD22 is a lectin-like adhesion receptor which has an important role to play in the regulation of B cell function and also forms part of the B cell activation complex [18]. As an anti-CD22 monoclonal antibody, epratuzumab can cause moderate depletion of B cells via ADCC; however, unlike rituximab, epratuzumab does not exhibit CDC or direct apoptosis of B cells [18]. Epratuzumab predominantly targets CD27^-^ B cells such as naive mature and transitional B cells and it is estimated that the reduction in peripheral B cell counts in SLE patients approximates 40% post-epratuzumab therapy [19].

EMBLEM™ is a 12-week, multi-center, randomized, double-blind, placebo-controlled, phase IIb study to assess the efficacy and safety of epratuzumab and determine a dose regimen in patients with moderate to severe SLE. A total of 227 patients were recruited and randomized to placebo n = 38, epratuzumab 200 mg cumulative dose (100 mg alternate weeks) n = 39, epratuzumab 800 mg cumulative dose (400 mg alternate weeks) n = 38, epratuzumab 2,400 mg cumulative dose (600 mg weekly) n = 37, epratuzumab 2,400 mg cumulative dose (1,200 mg alternate weeks) n = 37, epratuzumab 3,600 mg cumulative dose (1,800 mg alternate weeks) n = 38.

Epratuzumab at a cumulative dose of 2,400 mg was clinically effective and demonstrated a significant reduction in disease activity as measured by a composite disease activity score. Epratuzumab 600 mg weekly was associated with the greatest improvement in British Isles Lupus Assessment Group (BILAG)-2004 scores (from A/B to C/D) than placebo in all organ domains included in the study. Overall epratuzumab was well tolerated [18].

Two randomized controlled trials evaluating the efficacy of epratuzumab in severe SLE as determined by the presence of BILAG A (RCT SL0003) and/or moderate patients with BILAG B in at least two systems (RCT SL0004) were discontinued due to irregularities in the manufacture of epratuzumab. The results of patients recruited in these trials were pooled and indicate the potential benefit of epratuzumab in facilitating a reduction in prescribed corticosteroid dose [18].

Two Phase III, randomized, double-blind, placebo-controlled, multicenter studies of the efficacy and safety of four 12-week treatment cycles (48 weeks total) of epratuzumab in SLE subjects with moderate to severe disease EMBODY™1 & EMBODY™2 have an expected completion date of February 2014 with a recruitment of 780 patients. The main aim is to evaluate the efficacy, safety, tolerability and immunogenicity of epratuzumab in patients with moderate and severe SLE (NCT01262365, NCT01261793, http://www.clinicaltrials.gov). A phase III, multicenter, open-label, extension study to assess the safety and tolerability of epratuzumab treatment in SLE subjects EMBODY™4 started recruiting in July 2011 and is aiming to recruit 1,400 patients with a completion date of February 2016 (NCT01408576, http://www.clinicaltrials.gov).

### Ocrelizumab (anti-CD20)

Ocrelizumab is a humanized anti-CD20 monoclonal antibody. In 2010 an independent monitoring board recommended the suspension of clinical trials of ocrelizumab in rheumatoid arthritis and SLE due to a high frequency of reported severe and opportunistic infections in the patients enrolled in the trials. Therefore, the Study to Evaluate Ocrelizumab in Patients With Nephritis Due to Systemic Lupus Erythematosus (BELONG) trial was suspended [20].

The BELONG study had recruited 381 lupus nephritis class III and class IV patients to study the clinical efficacy and safety of ocrelizumab 400 mg or ocrelizumab 1,000 mg administered at baseline, a fortnight later, then every four months thereafter. All lupus nephritis patients enrolled in the study were treated with either intravenous cyclophosphamide using the EuroLupus regimen or MMF and high-dose corticosteroids concomitantly. Week 42 data from 221 patients who had enrolled at least 32 weeks prior to study termination have been reported in abstract form and, although ocrelizumab is clinically effective in reducing lupus nephritis disease activity, the data have not demonstrated superiority to standard immunosuppression [20].

### Targeting B-cell survival factors

#### Belimumab (anti-BLys)

Belimumab is a human immunoglobulin G1λ monoclonal antibody which blocks the binding of the soluble form of the cytokine B-lymphocyte stimulator (B-Lys), also known as B cell activating factor (BAFF), to the transmembrane activator/calcium modulator/cyclophilin ligand interactor (TACI) receptor, B-cell maturation (BCMA) receptor and BAFF receptor 3 (BR3) on B cells and thus interrupts the B cell survival role of B-Lys [21].

BAFF/BLys is expressed by several cells including dendritic cells, monocytes, activated neutrophils and T cells. It is vital in facilitating the maturation and survival of B cells via signaling through the BAFF-R, BCMA and TACI receptors with high, intermediate and low affinity respectively. APRIL, a BAFF homologue proliferation-inducing ligand binds with higher affinity to the TACI receptor than BAFF [22]. Dimerization of BAFF and APRIL to the BCMA receptor is required to support the maturation of plasma cells [22]. A strong interaction of BAFF to the BAFF-R propagates the maturation and survival of naive B cells and the interaction of BAFF/BLys, APRIL and TACI to the TACI-R facilitates immunoglobulin (Ig) gene class switching in the germinal center [22].

In the presence of an excess amount of BAFF/BLys, low-affinity self-reactive B cells may survive and mature into self-reactive auto-antibody secreting plasma cells implicated in autoimmune disease pathogenesis. As a result, it has been deduced that the inhibition of BAFF/BLys by belimumab has therapeutic implications in SLE.

In March 2011 the United States Food and Drug Administration (FDA) and the European Medicines Evaluation Agency (EMEA) licensed belimumab as the first new drug in over 50 years for SLE. Belimumab was licensed as a biologic agent to be prescribed with standard therapy for autoantibody-positive adult SLE patients excluding those with active lupus nephritis and central nervous system manifestations of SLE.

Belimumab is administered on a weight-based dosing schedule of belimumab 10 mg/kg as an hour long intravenous infusion fortnightly for three infusions then monthly thereafter.

A phase III randomized placebo-controlled trial Belimumab International SLE Study (BLISS-52) conducted between May 2007 and July 2009 included 865 SLE patients enrolled in Central and Eastern Europe, Latin America, and Asia Pacific [19]. A phase III randomized placebo-controlled trial Belimumab International SLE Study (BLISS-76) was conducted between February 2007 and February 2010 enrolling 819 patients in North America and Western and Central Europe [23]. These studies used the composite SRI outcome measure which requires improvement in the SELENA-SLEDAI but no worsening in the BILAG and Physician Global Assessment scores.

The trial outcome at 52 weeks in BLISS-52 reported positive clinical response in 44% of those treated with placebo with standard therapy, 51% of those treated with belimumab 1 mg/kg with standard therapy and 58% of those treated with belimumab 10 mg/kg with standard therapy (*P* = 0.013 and *P* = 0.0006, respectively) [23].

The trial outcome at 52 weeks in BLISS-76 reported positive clinical response in 34% of those treated with placebo with standard therapy, 41% of those treated with belimumab 1 mg/kg with standard therapy and 43% of those treated with belimumab 10 mg/kg with standard therapy (*P* = 0.10 and *P* = 0.021, respectively) [23]. However, at 76 weeks, there was no significant difference in responder rates between the belimumab and placebo groups.

The BLISS-52 and BLISS-76 clinical trials both excluded patients with active lupus nephritis. BLISS-LN is a phase III, randomized, double-blind, placebo-controlled study to evaluate the efficacy and safety of belimumab plus standard of care versus placebo plus standard of care in adult subjects with active lupus nephritis which will provide clinically relevant information about the use of belimumab in lupus nephritis NCT01639339 (http://www.clinicaltrials.gov).

An exploratory analysis of belimumab use in patients of black ethnicity in the BLISS-52 and BLISS-76 trials (n = 148) reported lower clinical effectiveness in this group as compared to other ethnic groups.

A phase III/IV multi-center, randomized, double-blind, placebo-controlled, 52-week study to evaluate the efficacy and safety of belimumab in adult subjects of black race with SLE is planned as a future study NCT01632241 (http://www.clinicaltrials.gov).

Belimumab may be more effective in specific sub-groups of lupus patients. Published data indicate that belimumab is significantly more efficacious in SLE patients who are ds-DNA positive, hypocomplementemic or have high disease activity as measured by SELENA-SLEDAI score >10 [24].

In 2012, fatal anaphylaxis was reported in a patient treated with belimumab and it is now known that there is a risk of a delayed acute hypersensitivity reaction to belimumab, especially in patients with multiple drug allergies. Long-term observational data will provide further safety and tolerability data on belimumab. At present the FDA Center for Drug Evaluation and Research has reviewed the safety labeling for belimumab (http://www.fda.gov/Safety/MedWatch/SafetyInformation/ucm299628).

The increased susceptibility to infection after belimumab treatment may be as a consequence of alterations in the signaling pathways involving BAFF/BLys and the TACI receptor. The TACI molecule has a complex role in host immunity involving activation of B cells and T cell independent immune regulation; however, this is yet to be completely understood [25]. In light of this, it has been postulated that the post-belimumab low BAFF/BLys levels result in a reduction in TACI signaling and hamper the host immune defenses against pathogens, such as polysaccharide encapsulated bacteria. Patients treated with belimumab have an increased susceptibility to infection, the commonest being pharyngitis, bronchitis, cystitis and viral gastroenteritis [23]. In the clinical trials serious infections have been reported in 6% of belimumab-treated patients as compared to 5.2% in placebo controls but there have been no reports to date of PML in belimumab treated patients [26].

Although belimumab received regulatory approval from the US FDA and the EMEA, its use in some countries has been restricted until approval by national drug evaluation organizations. The German Institute for Quality and Efficiency in Health Care (IQWiG) has recommended evaluation of belimumab for additional benefit over optimized immune-suppression rather than over standard therapy prior to full approval (http://www.iqwig.de).

In 2012 The National Institute for Health and Clinical Excellence (NICE) provided a draft national guidance on the use of belimumab for SLE in the United Kingdom. NICE did not recommend belimumab within its licensed indication as add-on therapy to standard immune-suppressive drugs in adult patients with active auto-antibody positive SLE. In making this decision, NICE considered the clinical trial evidence, clinical specialist and patient opinions. NICE concluded that the use of belimumab was not sufficiently cost-effective to the National Health Service (NHS) in relation to its reported clinical effectiveness. A final decision will be expected after the appeals process has been concluded (http://www.nice.org.uk).

### Blisibimod (anti-B-Lys)

In 2010 a Phase II study called PEARL-SC commenced with the aim of investigating the efficacy, safety, and tolerability of blisibimod, a B lymphocyte stimulatory antagonist, in patients with active SLE. In 2011 an open-label long-term safety extension trial for patients with SLE who completed the protocol PEARL-SC was commenced.

In 2012 approval was granted by the EMEA and FDA for phase III clinical trials of blisibimod, CHABLIS-SC1 and CHABLIS-SC2. These international multicenter, randomized, double-blind trials aim to evaluate the efficacy, safety, tolerability and immunogenicity of blisibimod in patients with severe active SLE (SELENA-SLEDAI >10) despite high-dose corticosteroids NCT01395745 (http://www.clinicaltrials.gov).

### Tabalumab (anti-B-Lys)

Tabalumab (LY2127399) is a human IgG4 monoclonal antibody targeting membrane-bound and soluble BAFF. A phase III, multicenter, randomized, double blind, placebo-controlled study to evaluate the efficacy and safety of subcutaneous LY2127399 in patients with SLE is expected to be completed in May 2015 (NCT01196091). Tabalumab is administered subcutaneously in addition to standard of care therapy for active SLE (http://www.clinicaltrials.gov).

### Atacicept (TACI-Ig fusion protein)

Atacicept is a TACI receptor fusion protein which inhibits BLys and APRIL in immature B cells, mature B cells and plasma cells. It is currently under investigation as a potential new therapy for SLE and is in a phase II/III clinical trial for patients with SLE excluding lupus nephritis [27]. The initial phase II trial of atacicept and MMF combination therapy for lupus nephritis was stopped due to a high frequency of reported infections likely related to a marked reduction in total Ig levels [28]. The prematurely terminated randomized, double-blind, placebo-controlled Phase II/III, 52-week study, APRIL-LN, reported adverse events in the patients randomized to atacicept (n = 4). Patients developed significant IgG hypogammaglobulinemia below the protocol-defined criteria for discontinuation (n = 3) and serious infections including, haemophilus influenza pneumonia, legionella pneumophilia pneumonia and bacillus bacteremia. Interestingly, atacicept trials in rheumatoid arthritis have not yielded this severity of adverse events [29]. This implies that the immunopathogenesis of lupus nephritis may have influenced the results of this atacicept trial.

### Blockade of T-cell co-stimulation

#### Abatacept (CTLA-4-Ig fusion protein)

Blockade of the co-stimulatory interactions between T and B lymphocytes can induce immunological tolerance. The most well characterized T lymphocyte co-stimulatory ligand is CD28, a glycoprotein which interacts with the co-stimulatory receptors B7-1 (CD80) and B7-2 (CD86). CTLA4 (cytotoxic T-lymphocyte antigen) is expressed on activated T cells and interacts with B7 with higher affinity than CD28 resulting in a negative feedback mechanism that inhibits T cell activation [30-32]. Abatacept is a fusion protein consisting of CTLA-4 combined with the Fc portion of human IgG1 (CTLA-4-Ig). Combination therapy of CTLA-4-Ig and cyclophosphamide significantly reduces proteinuria, autoantibody titres and improves mortality in murine lupus nephritis [33-35]. However, a randomized controlled trial of abatacept in 175 SLE patients failed to meet its primary end-point of a reduction of the proportion of patients with a new SLE flare [36]. Approximately one-fifth of the patients included in this study were sero-negative for ANAs and anti-dsDNA. There were, however some improvements in quality of life measures by the SF-36 physical component scores, fatigue and sleep problem scores in the abatacept treated group. Patients in this study primarily had musculoskeletal and dermatologic features of SLE and the trial was not specifically designed to examine the role of abatacept in lupus nephritis.

A 12-month Phase II/III double-blind placebo controlled trial in proliferative lupus nephritis failed to meet its primary end-point of time to complete renal response as defined as glomerular filtration rate within 10% of pre-flare/screening value, urinary protein creatinine ratio <0.26 mg/mg and inactive urinary sediment [37]. However when the same data were analyzed using different outcome measures, with complete response defined as serum creatinine either normal or ≤125% of baseline, urinary protein creatinine ratio <0.5 g/g, and prednisone dose ≤10 mg/d at study day 365, the study showed a positive outcome in favor of abatacept [38]. This highlights the importance of choosing outcomes measures in clinical trials of lupus nephritis and the necessity for standardization of outcomes across studies.

### Anti-CD40 ligand

CD40 ligand (CD40L) is a transmembrane glycoprotein belonging to the tumor necrosis factor (TNF) super family which binds with CD40 on the surface of B-cells and macrophages. The interaction between CD40/CD40L plays a pivotal role in B-cell class switching [39]. CD40L is over expressed in murine lupus models and monoclonal antibodies against CD40L have successfully treated murine lupus nephritis [40]. There have been two clinical trials of humanized anti-CD40L monoclonal antibodies (IDEC-131 and BG9588) in SLE patients. Eighty-five SLE patients treated with IDEC-131 failed to demonstrate clinical improvement as compared to placebo at 20 weeks [41]. A trial of 28 lupus nephritis patients treated with BG9588 showed initial promise with reduced anti-dsDNA titres and increasing complement levels but was discontinued prematurely due to unexpected thrombo-embolic side effects [42]. Given the lack of efficacy and toxicity demonstrated in these studies, it is unlikely that anti-CD40L will progress to larger clinical trials in SLE patients.

### Cytokine therapies

#### Tocilizumab (anti-IL-6)

IL-6 is a pleiotropic cytokine with both pro-inflammatory and anti-inflammatory properties and has been implicated in the pathogenesis of lupus nephritis. Exogenous IL-6 increases autoantibody production and accelerates progression of nephritis in both the NZB/NZW and BXSB lupus mouse models [43,44]. Treatment of lupus prone mice with an IL-6 monoclonal antibody decreases anti-dsDNA titres and proteinuria and reduces mortality [45,46]. In SLE patients, IL-6 levels have been shown to correlate with clinical activity and anti-dsDNA antibody levels [47,48]. Urinary excretion of IL-6 is increased in proliferative lupus nephritis and is reduced following cyclophosphamide therapy [49,50].

Tocilizumab is a fully humanized monoclonal antibody against the IL-6 receptor and prevents binding of IL-6 to both membrane bound and soluble IL-6 receptor. A phase I trial over a 12 week period has demonstrated the safety and tolerability of tocilizumab in SLE patients. While active urinary sediment and anti-dsDNA antibody titres were reduced, proteinuria remained unchanged [51]. The short duration of the study renders it difficult to draw conclusions as to the longer term effects of tocilizumab in the treatment of lupus nephritis. Randomized controlled trials of tocilizumab in SLE are awaited. Sirukumab (CNTO 136) a human monoclonal antibody that targets IL-6 is currently in a phase II study in lupus nephritis (NCT01273389) (http://www.clinicaltrials.gov).

### Targeting interferon-α

Recent studies of SLE patients and data from murine models of lupus, suggest that inappropriate activation of type I IFNs play an essential role in SLE pathogenesis. Microarray gene expression analysis has shown widespread activation of IFN-inducible genes in SLE patients which correlates with disease activity [52,53]. In addition, IFN pathway activation has been associated with lupus nephritis activity [54]. A scoring system based on expression of type I IFN-inducible mRNAs, which may divide SLE patients into two distinct subgroups has been proposed to enable type I IFN-inducible genes to be used as biomarkers to identify patients who might respond better to anti-type I IFN treatment [36]. Given the role of IFN-α in the host defense against viral infection, close clinical monitoring is mandatory in the development of any potential agents targeting this pathway.

Sifalimumab, a fully human anti-IFN-α monoclonal antibody, induced a dose-dependent inhibition of type I IFN-induced mRNAs (type I IFN signature) in whole blood in a phase I study. No increase in viral infections was noted and a general trend towards improvement in disease activity was seen [55]. Further studies examining the efficacy of sifalimumab in SLE are in recruitment (NCT01283139) (http://www.clinicaltrials.gov). A Phase II clinical trial evaluating rontalizumab, a recombinant humanized monoclonal antibody to IFN-α for SLE is also ongoing (NCT00962832) (http://www.clinicaltrials.gov).

The efficacy and safety of rontalizumab, a recombinant humanized monoclonal antibody to IFN-α was recently assessed in a randomized, double-blind, placebo-controlled phase II trial in adults with moderate to severe non-renal SLE. An abstract by Kalunian K *et al*. entitled ‘Efficacy and Safety of Rontalizumab (Anti-Interferon Alpha) in SLE Subjects with Restricted Immunosuppressant Use: Results of a Randomized, Double Blind, Placebo-Controlled Phase 2 Study’ was presented at the American College of Rheumatology Annual Scientific Conference in November 2012.

In the initial part of the study, SLE patients received either 750 mg intravenously of rontalizumab or placebo for four weeks. In the second part of the study, SLE patients received either 300 mg subcutaneously of rontalizumab or placebo for two weeks. Overall, response rates at 24 weeks as measured by BILAG and SRI were similar between rontalizumab and placebo. However, in patients taking >10 mg/kg of steroids daily, rontalizumab was more effective in reducing lupus disease activity than placebo. Patients were further analyzed as per their IFN gene expression signature, which showed that rontalizumab was more effective in those with a more elevated IFN signature.

### Complement therapies

#### Eculizumab (anti-C5)

The complement system plays an important role in the pathophysiology of SLE although individual complement components have distinct and varied functions in the disease process. Early components of the complement cascade are critical in the clearance of immune complexes and apoptotic material. Their absence in congenital C3 or C4 deficiency predisposes individuals to development of SLE. Activation of terminal complement components is associated with exacerbations of disease, particularly in lupus nephritis.

Monoclonal antibodies that specifically inhibit terminal complement activation while preserving early complement function have been developed. Eculizumab, a monoclonal antibody directed against the complement protein C5, inhibits the cleavage of C5 to C5a and C5b and thus blocks the formation of the terminal membrane attack complex C5b-9 [56]. Anti-C5 therapy delays onset of proteinuria, improves renal histology and survival in murine lupus nephritis [57]. A phase I trial of eculizumab in SLE demonstrated safety and tolerability, but no clear clinical improvements were seen by day 28 and 56 of the study [58]. To date there have been no further clinical trials to examine the potential efficacy of this therapy in SLE.

### Targeting Fcγ receptor IIB

Fcγ receptors are a heterogeneous group of hematopoietic cell surface glycoproteins that recognize the Fc portion of specific Ig isotypes, facilitating antibody-antigen interactions with effector cells and, thus, play a key role in the clearance of immune complexes [56]. Fcγ receptor IIB (FcγRIIB) is the sole inhibitory receptor in the Fcγ receptor family and competes with activatory Fcγ receptors expressed on immune cells for pathogenic immune complexes. FcγRIIB may also interfere with formation of memory/plasma cells that develop autoantibodies [56]. Treatment of lupus-prone NZB/NZW F1 mice with recombinant soluble FcγRIIB significantly delayed onset of proteinuria, reduced histopathological findings and improved survival [57]. Currently a soluble FcγRIIB (SM101) is undergoing phase II trials in SLE and primary immune thrombocytopenia (ITP).

### Laquinimod

Laquinimod is an oral quinoline-3-carboxamide small molecule which to date has been mainly investigated in the context of relapsing-remitting multiple sclerosis (MS). In MS, laquinimod biases the CD4+ phenotype in favor of Th2/Th3 cytokine production and inhibits disease development and infiltration of inflammatory cells into the CNS [58,59]. Laquinimod also suppresses major histocompatibility class II antigen presentation and down-regulates epitope spreading [60]. Laquinimod is currently in phase II trials in lupus arthritis and lupus nephritis. (http://www.clinicaltrials.gov).

### Janus kinase (JAK) and spleen tyrosine kinase (Syk) inhibitors

#### Tofacitinib (JAK inhibitor)

Tofacitinib is a Janus kinase (JAK) selective inhibitor which has been approved as the first oral biologic for the management of rheumatoid arthritis. JAKs are essential for signal transduction of cytokines and contribute to inflammatory responses [59]. Targeting JAKs in SLE would be a logical therapeutic option which can be studied further starting with trials to determine the safety, pharmacodynamics and efficacy of these drugs in SLE.

### Fostamatinib (Syk inhibitor)

Spleen tyrosine kinase (Syk) is implicated in the B cell immunopathogenesis of SLE and is a potential therapeutic target. Syk inhibitors have been shown to prevent the onset of skin and renal disease in lupus-prone mice. In addition, Syk inhibitors reduce inflammatory arthritis. Fostamatinib is an oral Syk inhibitor being evaluated for the management of autoimmune rheumatic diseases [60].

## Discussion

The management of SLE is likely to change significantly with the introduction of new biological therapies and the discovery of other therapeutic targets. The exact role of these drugs will be determined after completion of the trials and with clinical experience. It is envisioned that the majority of the biological therapies will initially be reserved for patients who have failed to respond satisfactorily to optimal conventional immunosuppressive drugs. The new biological drugs will need to be used appropriately to target disease remission; reduction of the disease severity; frequency of lupus flares and the subsequent high morbidity associated with lupus.

Conventional immunosuppressive therapies have radically transformed patient survival in SLE, but their use is associated with considerable toxicity and a substantial proportion of patients remain refractory to treatment. A more comprehensive understanding of the complexity of SLE immunopathogenesis has evolved over the past decade and has led to the testing of several biologic agents in clinical trials. An array of promising new therapies are yet to emerge or are under development. There is a clear need for new therapeutic strategies that overcome these issues, and biologic agents offer exciting prospects as future SLE therapies. The role of new therapeutic agents to date has chiefly centered on SLE patients who have been refractory to conventional therapies. There are few clinical trials examining their role as first line induction or maintenance therapy. Questions remain as to how these therapies can potentially be combined with existing proven treatments and indeed with one another to achieve maximum clinical benefit while minimizing toxicity. Although so far many biologics have been generally well tolerated, we must not be complacent regarding potential toxicity of these new agents, as we do not yet know the long-term effects of these medications on the immune system. Rituximab is currently used off-license for the management of severe refractory SLE and is likely to continue to be used for this indication due to overall positive clinical experience.

Based on the clinical trial and extension study data, belimumab has a modest level of clinical effectiveness when used in combination with standard immunosuppressive drugs in autoantibody-positive SLE patients. The BILAG data at week 52 of the BLISS trials suggested more favorable outcomes in the mucocutaneous, musculoskeletal domains. The SELENA-SLEDAI cutaneous, musculoskeletal, immunologic, vascular and CNS components significantly improved at week 52 in the BLISS trials. Physicians will, therefore, be inclined to closely monitor patients on belimumab and switch to alternate therapeutic regimens if the clinical response is inadequate after six months. SLE patients of black ethnicity are to be studied in greater numbers than in the original BLISS trials in order to ascertain whether or not belimumab is beneficial in this group of patients. As belimumab use becomes more prevalent and the results of the on-going belimumab clinical trials are published, the group of SLE patients likely to benefit the most from this drug may be identified and this will guide future use of this medication.

The place for other therapeutic agents in development for the management of SLE, such as epratuzumab, blisibimod, tabalumab and atacicept, as induction or maintenance therapies will be determined after robust reviews of the clinical trial data which are expected upon completion of the studies. It is anticipated that only drugs which show long-term clinical effectiveness, benefit as steroid-sparing agents and satisfactory safety profiles in SLE will gain approval for clinical use.

Some novel biologic therapies have been associated with significant toxicity leading to premature discontinuation of clinical trials such as the association of anti-CD40L and thrombo-embolic events and the high frequency of reported severe and opportunistic infections associated with ocrelizumab. Although some drugs have not progressed to phase II or III clinical trials after phase I studies, research into cytokine therapies, drugs targeting FcγRIIB and small molecule targets is on-going and may yield important results for the future of SLE management.

Health economic studies will be essential in determining the future use of the new therapeutic agents in SLE and may influence the international use of these drugs.

A number of key questions remain. How can these therapies be potentially combined with existing proven treatments and indeed with one another to achieve maximum clinical benefit with minimal side-effects, such as increased risk of serious infection. As is clear to all physicians involved in the day to day management of SLE patients, this is a heterogeneous disease and there is not one therapeutic regimen suitable for all. With a deeper understanding of the pathophysiology of SLE particularly from a genetic perspective, the era of personalized therapy may represent the greatest advance that is yet to come in optimizing treatment of SLE.

## Conclusions

Conventional immunosuppressive therapies have radically transformed patient survival in SLE, but their use is associated with considerable toxicity and a substantial proportion of patients remain refractory to treatment. A more comprehensive understanding of the complexity of SLE immunopathogenesis has evolved over the past decade and has led to the testing of several biologic agents directed against new molecular targets in clinical trials. An array of promising new therapies is yet to emerge or is under development. There is a clear need for new therapeutic agents that overcome these issues, and biologic agents offer exciting prospects as future SLE therapies. Several challenges still remain in designing clinical trials in SLE. One of the main issues is that conventional therapies have been optimized and are effective in the majority of patients. There is, therefore, quite a high bar for new therapies to demonstrate a significant benefit over conventional approaches and progress is likely to be incremental rather than revolutionary.

The role of new therapeutic agents has chiefly centered on SLE patients who have been refractory to conventional therapies. There are few clinical trials examining their role as first line induction or maintenance therapy. Questions remain around how these therapies can potentially be combined with existing proven treatments and indeed with one another to achieve maximum clinical benefit while minimizing toxicity. As is clear to all physicians involved in the day to day management of SLE patients, this is a heterogeneous disease and there is no single therapeutic regimen suitable for all. With a deeper understanding of the pathophysiology of SLE particularly from a genetic perspective, the era of personalized therapy may represent the greatest advance that is yet to come in optimizing treatment of SLE.

## Abbreviations

ADCC: Antibody-dependent cell toxicity; ANA: Anti-nuclear antibody; BAFF: B cell activating factor; BCMA: B cell maturation; BLys: B lymphocyte stimulator; BILAG: British Isles Lupus Assessment Group; CDC: Complement-dependent cell toxicity; CNS: Central nervous system; CTLA4: Cytotoxic T-lymphocyte antigen; dsDNA: Anti-double stranded DNA antibody; EMEA: European Medicines Evaluation Agency; ESRD: End-stage renal disease; FDA: United States Food and Drug Administration; IFN: Interferon; Ig: Immunoglobulin; IL-6: Interleukin-6; IL-10: Interleukin-10; IV: Intravenous; JAK: Janus kinase; MMF: Mycophenolate mofetil; PML: Progressive multifocal leuco-encephalopathy; RCT: Randomized controlled trials; SC: Subcutaneous; SELENA-SLEDAI: Safety of estrogens in lupus erythematosus national assessment trial systemic lupus erythematosus disease activity index; SF-36: Short form 36; SLE: Systemic lupus erythematosus; Syk: Spleen tyrosine kinase; SRI: Systemic lupus erythematosus responder index; TACI: Transmembrane activator/calcium modulator/cyclophilin ligand interactor; TNF: Tumor necrosis factor.

## Competing interests

NJ and PL have no competing interests. DDC has received consulting fees and/or has participated in clinical trials for Glaxo-SmithKline, Bristol Myers Squibb, Roche and Eli-Lily.

## Authors’ information

NJ joined the lupus team at St Thomas’ Hospital in 2009 and was subsequently awarded an Arthritis Research UK clinical research fellowship to undertake a PhD focusing on lupus nephritis. Her research is based at the Centre for Molecular and Cellular Biology of Inflammation at King’s College London and she continues to work as a clinician at the Louise Coote Lupus Unit primarily in the areas of lupus nephritis and vasculitis. PL joined the lupus team at St Thomas’ Hospital in 2010 as a specialist registrar in Rheumatology and is currently a clinical research fellow at the Peter Gorer Department of Immunobiology King’s College London and Lupus Unit St Thomas’ Hospital studying the effects of B cell depletion therapy on lymphocyte subsets in lupus and vasculitis. DDC is clinical team lead for the Louise Coote Lupus Unit and is Professor of Lupus Biology and Consultant Rheumatologist.

## Pre-publication history

The pre-publication history for this paper can be accessed here:

http://www.biomedcentral.com/1741-7015/11/120/prepub
